# Cancer type prediction based on copy number aberration and chromatin 3D structure with convolutional neural networks

**DOI:** 10.1186/s12864-018-4919-z

**Published:** 2018-08-13

**Authors:** Yuchen Yuan, Yi Shi, Xianbin Su, Xin Zou, Qing Luo, David Dagan Feng, Weidong Cai, Ze-Guang Han

**Affiliations:** 1 Key Laboratory of Systems Biomedicine, Shanghai Center for Systems Biomedicine, Shanghai Jiaotong University, Shanghai, 200240 China; 2 School of Information Technologies, University of Sydney, Sydney, NSW 2006 Australia

**Keywords:** Copy number aberration, HiC, Somatic mutation, Cancer type prediction, Deep learning, Convolutional neural network

## Abstract

**Background:**

With the developments of DNA sequencing technology, large amounts of sequencing data have been produced that provides unprecedented opportunities for advanced association studies between somatic mutations and cancer types/subtypes which further contributes to more accurate somatic mutation based cancer typing (SMCT). In existing SMCT methods however, the absence of high-level feature extraction is a major obstacle in improving the classification performance.

**Results:**

We propose DeepCNA, an advanced convolutional neural network (CNN) based classifier, which utilizes copy number aberrations (CNAs) and HiC data, to address this issue. DeepCNA first pre-process the CNA data by clipping, zero padding and reshaping. Then, the processed data is fed into a CNN classifier, which extracts high-level features for accurate classification. Experimental results on the COSMIC CNA dataset indicate that 2D CNN with both cell lines of HiC data lead to the best performance. We further compare DeepCNA with three widely adopted classifiers, and demonstrate that DeepCNA has at least 78% improvement of performance.

**Conclusions:**

This paper demonstrates the advantages and potential of the proposed DeepCNA model for processing of somatic point mutation based gene data, and proposes that its usage may be extended to other complex genotype-phenotype association studies.

## Background

Cancer is a category of disease that causes abnormal cell growths and immortality. It usually incarnates into a tumor form that potentially invades or metastasizes to remote parts of the human body [1]. Cancer is known as one of the major lethal diseases that leads to about 8.2 million, or 14.6%, of all human deaths each year [2]. Considerable research endeavors, therefore, have been devoted to cancer diagnosis and therapy techniques to alleviate the impact of cancer to human health, among which, somatic mutation based cancer typing (SMCT) is one of the most important research topics. SMCT aims to determine the cancer types/subtypes based on a patient’s somatic gene mutations, so that a therapy plan can be made accordingly. As the cost of DNA sequencing has dropped in recent years, there has been a dramatic increase in DNA sequencing data, which promotes the developments of SMCT to a large extent [3].

Unlike the conventional cancer typing methods that are usually based on morphological appearances or gene expression levels (i.e., mRNA profiles or protein profiles) of the tumor, SMCT is able to differentiate tumors that have similar histopathological appearances [4], which makes it significantly more robust to environmental influences, and more favorable in delivering accurate tumor typing results. There are several types of somatic DNA mutations, namely point mutation (or single nucleotide variation, SNV), small insertion and deletion (INDEL), copy number aberration (CNA), and translocation. They have all been shown to be associated with different cancers [5, 6]. We have previously demonstrated the cancer type prediction capacity of deep learning using point mutation alone [7]. In this work, we aim to investigate how CNAs contribute to cancer type prediction. This exploration has the following significances.

1. The link between aneuploidy and cancer has long been recognized over a century ago [8], and are attracting more attentions in recent years [9]. Known as one of the principle contributors to genetic heterogeneity in cancer and an important determinant of clinical prognosis and therapeutic resistance [10], chromosomal instability (CIN) is a process in which CNAs arise from persistent errors in chromosome segregation during cell division.
2. As the major form of chromosomal instability, CNAs affect a larger fraction of the genome in cancers than any other type of somatic genetic alteration [11], and is critical in activating oncogenes and inactivating tumor suppressors [12–14]. For example, genomic imbalances have been found in 5918 epithelial tumors [15]. Stephens et al. identified somatic CNA in breast cancer genomes and found that there were more rearrangements in some breast cancers than previously appreciated [16].
3. The technologies of profiling genome-wide CNV are more developed than before, from DNA microarray based [17] to whole-genome DNA sequencing based [18] to exome sequencing based [19], and the cost is dropping in a Moore’s law fashion. Therefore, the combinatorial CNA patterns learned in predicting cancer types/subtypes can be easily used for developing cost-effective diagnosis CNA marker panels.

Clinically, SMCT may significantly facilitate cancer-related diagnoses and treatments, such as personalized tumor medicine [4], targeted tumor therapy [5] and compound medicine [20]. It can also aid cancer early diagnosis (CED) in combination with the sampling and sequencing of circulating tumor cells (CTCs) or circulating DNA (ctDNA) [6].

Over the past two decades, the boom of machine learning techniques has facilitated the researches in bioinformatics to a large extend, including SMCT. In order to predict the cancer types/subtypes more effectively, many machine learning approaches have been proposed in existing cancer type prediction studies, which have exhibited promising results [21–24]. For instance, remarkable developments have been demonstrated in tumor cases of colorectal [25], breast [26], brain [27], and melanoma [28]. However, there are still major, unresolved challenges. More specifically, different genes related to specific types of cancer are generally correlated and have complex interactions which may impede the application of conventional simple linear classifiers such as linear kernel support vector machine (SVM) [29]. Therefore, it is desirable to devise an advanced classifier capable of extracting high level features within the discriminatory subset. Although there have been recent works utilizing sparse-coding [30] or auto-encoder for gene annotation, no work has been devoted to applying high-level machine learning approaches to SMCT [7].

In recent years, the developments of deep neural network (DNN) [31] have equipped bioinformaticians with powerful machine learning tools. DNN is a type of artificial neural network that aims to model abstracted high-level data features using multiple nonlinear and complex processing layers, and provides feedback via back-propagation [32]. First introduced in 1989 [33], DNN has garnered tremendous developments and is widely applied in image classification [34], object localization [35], facial recognition [36], and saliency detection [37] etc. DNN has the potential to introduce novel opportunities for SMCT where it perfectly fits the need for large scale data processing and high level feature extraction. However, to the present, applying customized DNN on SMCT is yet to be explored.

In this paper, we propose a novel SMCT method, named DeepCNA, designed to address the absence-of-high-level-feature issue above. DeepCNA is a DNN-based classification model composed of two steps. It first conducts several novel pre-processing steps on the CNA data, which includes data clipping, zero padding, and data reshaping; after the first step, the CNA data is formulated in matrix format so that the subsequent machine learning techniques such as convolutional neural network (CNN) can be applied in predicting the cancer type of the target sample. Since 2009, Lieberman et al. [38] developed the HiC technology that can capture the high order chromatin conformation genome-wide; considering the CNAs can be intrinsically linked to each other in the context of chromatin 3D structure, we adopt the HiC data into our DeepCNA pipeline as well.

## Methods

### Data preprocessing

Before conducting any experiments with the neural networks, the CNA data needs to be preprocessed and standardized. In our proposed method, three steps are conducted as preprocessing:

1. The CNA data is first empirically clipped into the interval [0, 10], which regulates the data values into desired range and dismiss extremely large values that may impede the training.
2. The clipped data is then zero-padded at tail to have the desired length that fits the input of the subsequent neural networks, which produces 1*1 features maps before the fc layers. Since our raw CNA data has 29,915 features, for 1D CNN, 2853 zeros are padded to make the CNA sample has the length 32,768 (29,915 + 2853 = 32,768); while for 2D CNN, 1061 zeros are padded to make the sample has the length 176*176 (29,915 + 1061 = 176*176).
3. For 2D CNN, the CNA samples are then reshaped into 176*176*1, just like single-layered images.

### 1D convolutional neural network

We first try the 1D CNN, which consists of multiple 1D convolutional layers. Compared with fully connected networks, our 1D CNN takes into account the local correlations of different features, which significantly facilitates high-level feature extraction. Moreover, the weight sharing of CNN is able to drastically lower the degree of freedom of the network, and thus reduce its overall size, making deeper networks practical.

The architecture of our 1D CNN is shown in Table 1. It is a feed-forward neural network trained by back-propagation [33]. The number of input channels depends on whether the HiC data is used, i.e. if the HiC data is adopted, they will be appended to the CNA data as additional input layers. There are 6 convolutional layers and 2 fully connected layers established as hidden layers for data processing, together with ReLU [39] as the activation function, and max pooling for progressive spatial size reduction. A softmax function is applied after fc8 to convert its outputs into probabilities, which are then fed into the loss layer for logarithm loss computation. The output number is determined by the number of total cancer types; which is 25 in our case.

**Table 1:** Architecture of our proposed 1D CNN

Layer	Type	Output size	Conv (size, channel, pad)	Max pooling
input	in	32768*1*ch	N/A	N/A
conv1	c + r + p	8192*1*32	3*1, 32, 1	4*1
conv2	c + r + p	2048*1*64	3*1, 64, 1	4*1
conv3	c + r + p	512*1*128	3*1, 128, 1	4*1
conv4	c + r + p	128*1*256	3*1, 256, 1	4*1
conv5	c + r + p	32*1*512	3*1, 512, 1	4*1
conv6	c + r	1*1*4096	32*1, 4096, 0	N/A
fc7	fc + r + d	1*1*4096	1*1, 4096, 0	N/A
fc8	fc	1*1*25	1*1, 25, 0	N/A
loss	sm + log	1*1	N/A	N/A

Annotations - in: input layer; c: convolutional layer; r: ReLU layer; p: pooling layer; fc: fully connected layer; d: dropout layer; sm: softmax layer; log: log loss layer; ch: number of input channels (depending on whether the HiC data is used); asterisk(*): multiplication

Unlike conventional DNN classifiers for 1D data that entirely based on the bulky fully connected layers [7], our 1D CNN introduces 1D convolution that effectively exploits correlations among local data with shared weights, which significantly reduces the overall size of the network, and makes it practical for deeper and more powerful networks for 1D input data. The resulting deeper networks will thus offer better performance in the high-level feature extraction of the CNA data, and lead to higher accuracy in the cancer type classification.

### 2D convolutional neural network

Although the 1D CNN introduced in section 0 can potentially improve the classification accuracy, further exploitation of the CNN’s capacity in high level feature extraction can still be explored. The great success of the recently prevalent 2D CNN on image classification tasks [34, 40] suggests a highly promising way for 1D data classification, i.e. convert the 1D data into image-like matrices and apply the 2D convolution. Compared with 1D convolution, the 2D convolution is able to analyze the pattern of the data in a larger picture beyond the immediate local perspective, introducing potential correlations from broader ranges of the data.

The CNA data vector and its corresponding HiC data are reshaped into 176*176 before put into the network. The architecture of our 2D CNN is shown in Table 2. Similar to the 1D CNN, it also consists of multiple convolutional layers for feature extraction, ReLU as activation function, and max pooling for progressive spatial size reduction.

**Table 2:** Architecture of our proposed 2D CNN

Layer	Type	Output size	Conv (size, channel, pad)	Max pooling
input	in	176*176*ch	N/A	N/A
conv1	c + r + p	88*88*32	3*3, 32, 1	2*2
conv2	c + r + p	44*44*64	3*3, 64, 1	2*2
conv3	c + r + p	22*22*128	3*3128, 1	2*2
conv4	c + r + p	11*11*256	3*3, 256, 1	2*2
conv5	c + r	1*1*1024	11*11, 1024, 0	N/A
fc6	fc + r + d	1*1*1024	1*1, 1024, 0	N/A
fc7	fc	1*1*25	1*1, 25, 0	N/A
loss	sm + log	1*1	N/A	N/A

Annotations - in: input layer; c: convolutional layer; r: ReLU layer; p: pooling layer; fc: fully connected layer; d: dropout layer; sm: softmax layer; log: log loss layer; ch: number of input channels (depending on whether the HiC data is used); asterisk(*): multiplication

## Results

### Dataset

Our experiments are all conducted on the newly proposed COSMIC CNA dataset [41]. After disposition, we obtain a CNA matrix *C*, which has the dimension 14,703 samples by 29,915 genes that covers 25 cancer types (primary sites). An element *c*_*ij*_ in *C* indicates the somatic copy number of sample *i* in gene *j*. To deal with outlier issues, all the copy numbers that are greater than 10 are clipped into 10. For the chromatin 3D structure data, we adopt HiC data of two human cell lines, hESC and IMR90, with resolution 40 KB and 500 KB from Bin Ren’s lab [42].

### Constant parameters

For both the 1D CNN and the 2D CNN, the output size of their loss layer is set to 25, which is equal to the number of cancer types to be classified. As for the network parameters, the total training iteration is set to 20,000; the base learning rate is set to 0.001, which shrinks by 10 fold for each 5000 iterations; the weight decay is set to 0.002; and the training batch size is set to 200.

### Evaluation metrics

In all of our experiments, we adopt the 10-fold cross validation accuracy as our evaluation metric for the performance. To make the comparison among different methods fair, the same data division is used. The dataset is randomly divided into 10 equal subgroups, and for each fold of the cross validation, 90% (13,222) of the samples are used for training, while the rest 10% (1481) for testing.

### Implementation

Both the 1D CNN and the 2D CNN are implemented in Python under the Caffe framework [43], which is an open source framework for CNN training and testing. The machine used for our experiments is a PC with Intel 6-Core i7-5820 K 3.3GHz CPU, 64GB RAM, GeForce GTX TITAN X 12GB GPU, and 64-bit Ubuntu 14.04.3 LTS. Software dependencies include CUDA 8.0 and cuDNN 5.1.

### Evaluation of design options

We first evaluate the impact of the HiC data. To keep consistency, both resolution settings (40 KB and 500 KB) of the two cell lines of HiC data (hESC and IMR90) are always used together. In general, there are four possible combinations:

1. No HiC data (1 channel);
2. hESC only (3 channels);
3. IMR90 only (3 channels); and
4. both hESC and IMR90 (5 channels).

**Fig. 1:**
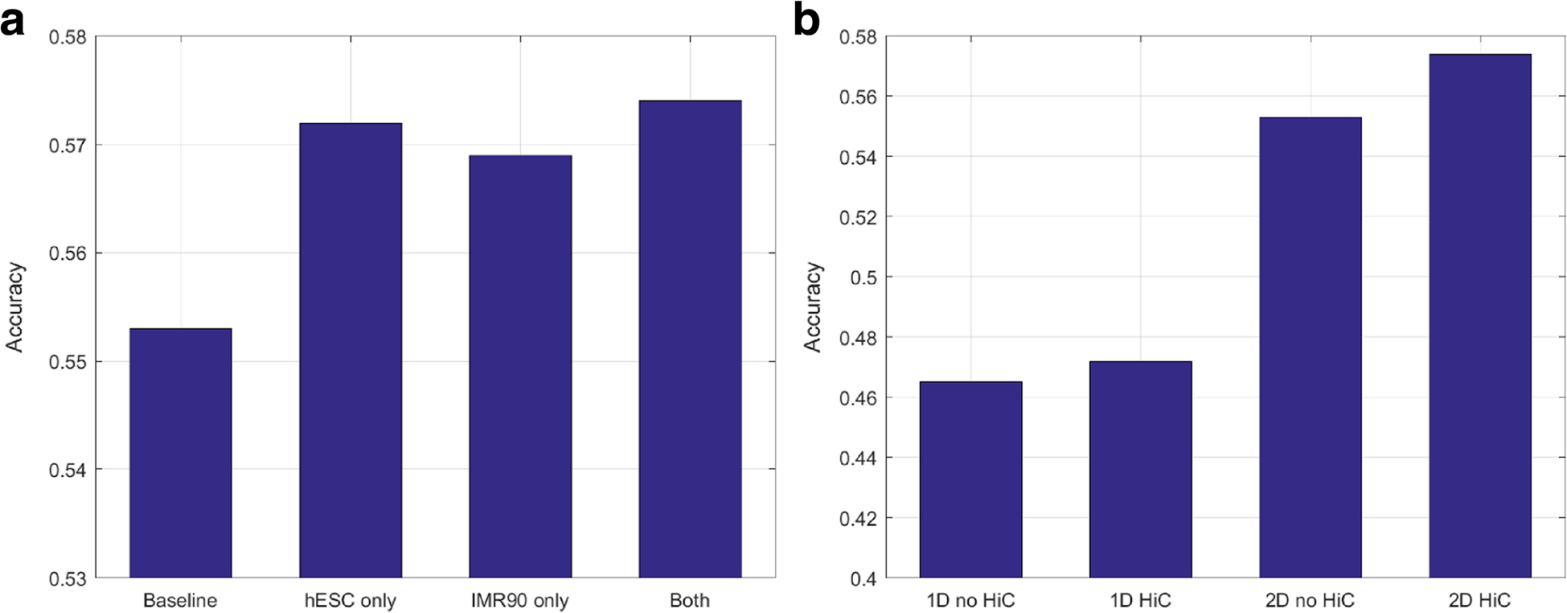
Performances of our proposed method with different design options. **a** With different HiC data configurations. From left to right: baseline model (2D CNN); baseline with hESC only; baseline with IMR90 only; baseline with both types of HiC data. The last configuration leads to the optimal performance. **b** With different network and HiC combinations. From left to right: 1D CNN without HiC data; 1D CNN with HiC data; 2D CNN without HiC data; 2D CNN with HiC data. The last configuration leads to the optimal performance

The number in the parenthesis indicates the number of input channels to the CNN. We use the 2D CNN as the baseline model (1). The performances of the four configurations above are shown in Fig. 1a. It is observed that configuration (4) outperforms the other three configurations, and is thus adopted in our following experiments.

After that, we evaluate the impact of the two major design options in our proposed method, namely the network architecture (1D or 2D CNN), and the usage of HiC data (whether or not the HiC data is used). This includes four possible configurations:

1. 1D CNN without HiC data (1 channel);
2. 1D CNN with HiC data (5 channels);
3. 2D CNN without HiC data (1 channel); and
4. 2D CNN with HiC data (5 channels).

The number in the parenthesis indicates the number of input channels to the CNN. The performances of the four configurations above are shown in Fig. 1b. It is apparent that the 2D CNN with HiC data leads to the highest performance. This configuration hence determines the final model of our proposed DeepCNA method.

### Evaluation against widely adopted methods

To compare our proposed DeepCNA method against the state-of-the-art, we select three most representative data classifiers that are prevalently used in gene-based cancer type classifications, namely support vector machine (SVM) [29], *k-*nearest neighbors (KNN) [44], and naïve Bayes (NB) [45]. All of the three comparison methods are implemented with the sklearn toolbox of Python. To conduct fair evaluation against DeepCNA, the comparison methods use raw CNA data (without HiC) as input, and the 10-fold cross validation accuracy as evaluation metric as well. We set up the parameters of the three comparison methods as below.

- SVM: we test three different kernel types, namely linear, polynomial (degree = 3) and RBF, while keep all of the other parameters as default.
  Table 3 shows the performances with different kernels, in which the polynomial kernel leads to the best result.
- KNN: we alternatively change the number of neighbors and the *p* value, and keep all the other parameters as default. The performances are recorded in Table 4. It is observed that n_neighbors = 5 and *p* = 2 lead to the optimal performance.
- NB: we test three different types of data distribution assumptions, namely Bernoulli, multinomial and Gaussian. The performances are recorded in Table 5. Based on the results, the multinomial distribution contributes to the best performance.

**Table 3:** Evaluation of SVM with different kernel types

Kernel	Linear	Polynomial	RBF
Accuracy	0.317	0.322	0.275

**Table 4:** Evaluation of KNN with different number of neighbors and p value

p\*n*_neighbors	3	4	5	6	7
1	0.257	0.259	0.262	0.265	0.266
2	0.263	0.273	0.283	0.279	0.277
3	0.254	0.259	0.264	0.258	0.262

**Table 5:** Evaluation of NB with different data distribution assumptions

Distribution	Bernoulli	Multinomial	Gaussian
Accuracy	0.161	0.238	0.139

**Fig. 2:**
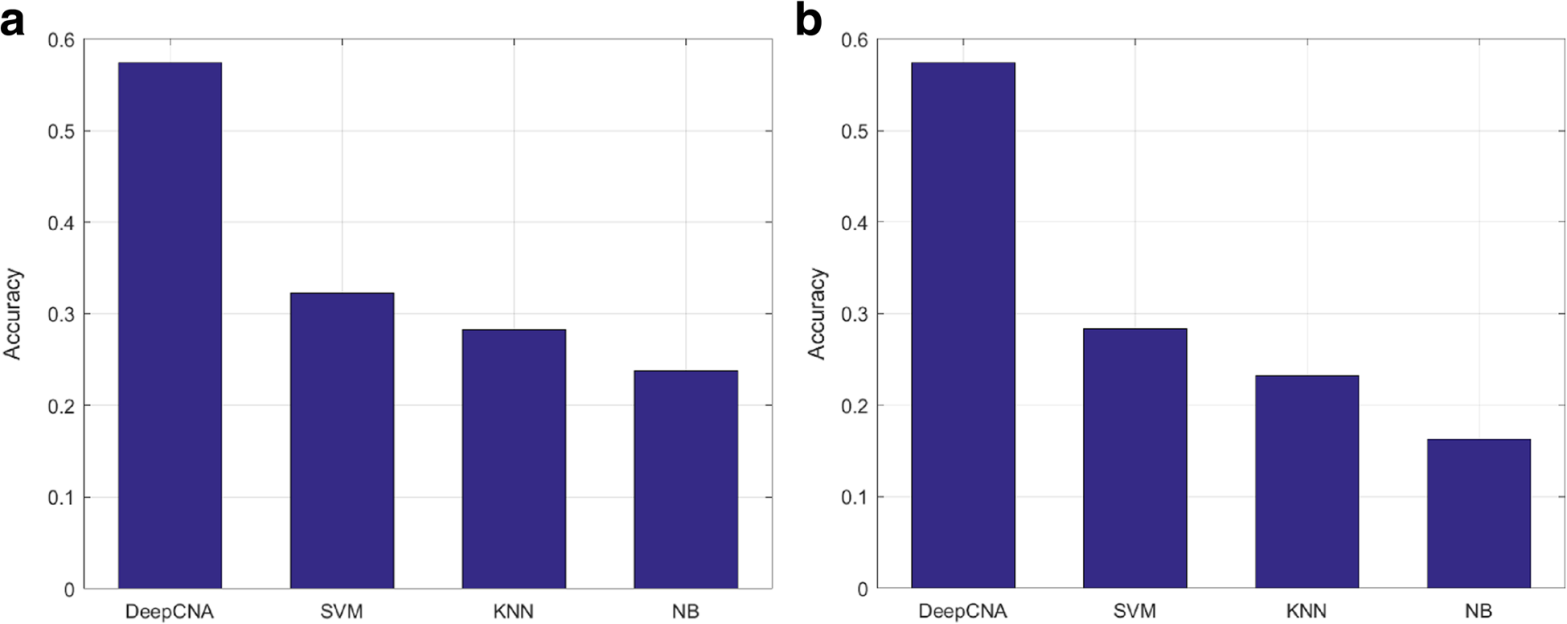
Performances of our proposed method against three widely adopted data classifiers. **a** The comparison methods use raw CNA input data (without HiC). From left to right: Our method, SVM (polynomial kernel), KNN (number of neighbors = 5 and *p* = 2) and NB (multinomial distribution). Our method shows significant advantage against the comparison methods. **b** The comparison methods use both CNA and HiC as input data. From left to right: Our method, SVM (polynomial kernel), KNN (number of neighbors = 5 and p = 2) and NB (multinomial distribution). Our method shows even greater advantage against the comparison methods

We then proceed to the experiment between DeepCNA and the comparison methods, the results of which are plotted in Fig. 2a. Our method exhibits dominant advantage against all of the three comparison methods. The performance improvements are 78.3% (0.574 vs. 0.322), 103% (0.574 vs. 0.283) and 141% (0.574 vs. 0.238) against SVM, KNN and NB, respectively.

To further evaluate the effectiveness of HiC, we re-evaluate the comparison methods, but add the HiC data to the input. Considering that the three comparison models take 1D data as samples, we reshape the HiC data into 1D, which is subsequently concatenated to the tail of the raw CNA data. The new results are plotted in Fig. 2b. Contrary to intuition, however, the performances of the comparison methods get worse with the HiC data concatenated. Their accuracies have dropped by 11.8% (0.284 vs. 0.322), 18.0% (0.232 vs. 0.283) and 31.5% (0.163 vs. 0.238) for SVM, KNN and NB, respectively.

## Discussion

The results in Fig. 2 clearly exhibit the dominant advantage of DeepCNA against the three widely adopted comparison methods. We attribute the success of our method to its utilization of the CNN, and especially the convolutional layers in the network.

Conventional 1D data classification methods mainly rely on classic machine learning classifiers (e.g. SVM) [46, 47] or fully connected neural networks [7], which conduct data classification without the use of high level features. These methods perform well on small-scaled samples, but encounter difficulty on large-scaled samples, such as the data in our experiments. On the other hand, the weight sharing of the convolutional layers significantly reduces the overall size of CNN, which greatly facilitates the establishment of deeper and more powerful neural network architectures. The deeper networks may effectively extract the high level features within the large-scaled input data, leading to higher performances in the classification tasks.

It is also notable that due to the intrinsic limitations, the classic machine learning classifiers do not always offer higher performances as the feature number of the input increases. This is evidenced in Fig. 2b, where the introduction of the HiC data deteriorates the accuracies of the three comparison methods, unlike the case in our method where HiC data improves the performance.

One potential extension of this work relies on incorporating heterogeneous data sources, such as somatic point mutation, small insertion and deletion, chromatin translocation, DNA methylation, gene expression, as well as copy number aberration. This requires high quality samples which contain as many heterogeneous data sources as possible.

## Conclusions

In this paper, we propose the DeepCNA method for SMCT. DeepCNA consists of two major steps. The pre-processing step regulates the CNA data with clipping, zero padding, and reshaping; while the CNN step takes the pre-processed data and generates the classification result with high-level data feature learning.

We conduct experiments on the newly proposed COSMIC CNA dataset, which contains 25 types of cancer. Controlled variable experiments indicate that the 2D CNN with both cell lines of HiC data (hESC and IMR90) contributes to the optimal performance. We believe that HiC data brings the gene spatial information such as co-localization into the deep learning model, and due to the possibility that co-localized genes may have similar CNV profiles, combining these two types of information into the predictor improves the overall prediction power as they cross-validate to each other.

We then compare DeepCNA with three widely adopted data classifiers, the results of which exhibit the remarkable advantages of DeepCNA, which has achieved significant performance improvements in terms of testing accuracy against the comparison methods.

We have demonstrated the advantages and potentials of the DeepCNA model for somatic point mutation based gene data processing, and suggest that the model can be extended and transferred to other complex genotype-phenotype association studies, which we believe will benefit many related areas. As for future studies, we will refine our model for other complex and large-scale data, as well as broadening our training dataset, so that the classification result can be further improved.

## Abbreviations

CED: Cancer early diagnosis
CNA: Copy number aberration
CNN: Convolutional neural network
CTC: Circulating tumor cell
ctDNA: Circulating DNA
SMCT: Somatic mutation based cancer typing
SNV: Single nucleotide variation

## References

[1] Feuerstein M (2007). Defining cancer survivorship. J Cancer Surviv.

[2] Stewart B, Wild CP (2015). “World cancer report 2014,” World.

[3] Golub TR, Slonim DK, Tamayo P, Huard C, Gaasenbeek M, Mesirov JP (1999). Molecular classification of cancer: class discovery and class prediction by gene expression monitoring. Science.

[4] Longo DL (2012). Tumor heterogeneity and personalized medicine. N Engl J Med.

[5] Sledge GW (2005). What is targeted therapy?. J Clin Oncol.

[6] Franken B, de Groot MR, Mastboom WJ, Vermes I, van der Palen J, Tibbe AG (2012). Circulating tumor cells, disease recurrence and survival in newly diagnosed breast cancer. Breast Cancer Res.

[7] Yuan Y, Shi Y, Li C, Kim J, Cai W, Han Z (2016). DeepGene: an advanced cancer type classifier based on deep learning and somatic point mutations. BMC Bioinformatics.

[8] Boveri T. Ueber mehrpolige mitosen als mittel zur analyse des zellkerns, Vehr d phys med Ges zu Wurzburg N. 1902;35:67–90.

[9] Bakhoum SF, Swanton C (2014). Chromosomal instability, aneuploidy, and cancer. Front Oncol.

[10] Burrell RA, McGranahan N, Bartek J, Swanton C (2013). The causes and consequences of genetic heterogeneity in cancer evolution. Nature.

[11] Zack TI, Schumacher SE, Carter SL, Cherniack AD, Saksena G, Tabak B (2013). Pan-cancer patterns of somatic copy number alteration. Nat Genet.

[12] Beroukhim R, Mermel CH, Porter D, Wei G, Raychaudhuri S, Donovan J (2010). The landscape of somatic copy-number alteration across human cancers. Nature.

[13] Kim T-M, Xi R, Luquette LJ, Park RW, Johnson MD, Park PJ (2013). Functional genomic analysis of chromosomal aberrations in a compendium of 8000 cancer genomes. Genome Res.

[14] Nik-Zainal S, Van Loo P, Wedge DC, Alexandrov LB, Greenman CD, Lau KW (2012). The life history of 21 breast cancers. Cell.

[15] Baudis M (2007). Genomic imbalances in 5918 malignant epithelial tumors: an explorative meta-analysis of chromosomal CGH data. BMC Cancer.

[16] Stephens PJ, McBride DJ, Lin M-L, Varela I, Pleasance ED, Simpson JT (2009). Complex landscapes of somatic rearrangement in human breast cancer genomes. Nature.

[17] Carter NP (2007). Methods and strategies for analyzing copy number variation using DNA microarrays. Nat Genet.

[18] Wang H, Nettleton D, Ying K (2014). Copy number variation detection using next generation sequencing read counts. BMC Bioinformatics.

[19] Sathirapongsasuti JF, Lee H, Horst BAJ, Brunner G, Cochran AJ, Binder S (2011). Exome sequencing-based copy-number variation and loss of heterozygosity detection: ExomeCNV. Bioinformatics.

[20] Gudeman J, Jozwiakowski M, Chollet J, Randell M (2013). Potential risks of pharmacy compounding. Drugs in R&D.

[21] Yang K, Li J, Cai Z, Lin G (2005). A model-free and stable gene selection in microarray data analysis. Bioinformatics and Bioengineering, 2005. BIBE 2005. Fifth IEEE Symposium on.

[22] Yang K, Cai Z, Li J, Lin G (2006). A stable gene selection in microarray data analysis. BMC Bioinformatics.

[23] Cai Z, Goebel R, Salavatipour MR, Lin G (2007). Selecting dissimilar genes for multi-class classification, an application in cancer subtyping. BMC Bioinformatics.

[24] Cai Z, Zhang T, Wan X-F (2010). A computational framework for influenza antigenic cartography. PLoS Comput Biol.

[25] Huang Z, Huang D, Ni S, Peng Z, Sheng W, Du X (2010). Plasma microRNAs are promising novel biomarkers for early detection of colorectal cancer. Int J Cancer.

[26] Aaroe J, Lindahl T, Dumeaux V, Saebo S, Tobin D, Hagen N (2010). Gene expression profiling of peripheral blood cells for early detection of breast cancer. Breast Cancer Res.

[27] Balss J, Meyer J, Mueller W, Korshunov A, Hartmann C, von Deimling A (2008). Analysis of the IDH1 codon 132 mutation in brain tumors. Acta Neuropathol.

[28] Winnepenninckx V, Lazar V, Michiels S, Dessen P, Stas M, Alonso SR (2006). Gene expression profiling of primary cutaneous melanoma and clinical outcome. J Natl Cancer Inst.

[29] Cortes C, Vapnik V (1995). Support-vector networks. Mach Learn.

[30] Harrow J, Frankish A, Gonzalez JM, Tapanari E, Diekhans M, Kokocinski F (2012). GENCODE: the reference human genome annotation for the ENCODE project. Genome Res.

[31] Hinton GE, Salakhutdinov RR (2006). Reducing the dimensionality of data with neural networks. Science.

[32] Deng L, Yu D (2014). Deep learning: methods and applications. Foundations and Trends in Signal Processing.

[33] LeCun Y, Boser B, Denker JS, Henderson D, Howard RE, Hubbard W (1989). Backpropagation applied to handwritten zip code recognition. Neural Comput.

[34] Szegedy C, Liu W, Jia Y, Sermanet P, Reed S, Anguelov D, et al. Going deeper with convolutions. Cornell University library; 2014 arXiv preprint arXiv:1409.4842.

[35] Long J, Shelhamer E, Darrell T. Fully convolutional networks for semantic segmentation. Cornell University library; 2014 arXiv preprint arXiv:1411.4038. 10.1109/TPAMI.2016.2572683 27244717

[36] Sun Y, Wang X, Tang X (2014). Deep learning face representation from predicting 10,000 classes. Computer Vision and Pattern Recognition (CVPR), 2014 IEEE Conference on.

[37] Yuan Yuchen, Li Changyang, Kim Jinman, Cai Weidong, Feng David Dagan (2018). Dense and Sparse Labeling With Multidimensional Features for Saliency Detection. IEEE Transactions on Circuits and Systems for Video Technology.

[38] Lieberman-Aiden E, Van Berkum NL, Williams L, Imakaev M, Ragoczy T, Telling A (2009). Comprehensive mapping of long-range interactions reveals folding principles of the human genome. Science.

[39] Nair V, Hinton GE (2010). Rectified linear units improve restricted boltzmann machines. Proceedings of the 27th international conference on machine learning (ICML-10).

[40] Simonyan K, Zisserman A. Very deep convolutional networks for large-scale image recognition. Cornell University library; 2014 arXiv preprint arXiv:1409.1556.

[41] Forbes SA, Beare D, Gunasekaran P, Leung K, Bindal N, Boutselakis H (2015). COSMIC: exploring the world's knowledge of somatic mutations in human cancer. Nucleic Acids Res.

[42] Dixon JR, Selvaraj S, Yue F, Kim A, Li Y, Shen Y (2012). Topological domains in mammalian genomes identified by analysis of chromatin interactions. Nature.

[43] Jia Y, Shelhamer E, Donahue J, Karayev S, Long J, Girshick R (2014). Caffe: convolutional architecture for fast feature embedding. Proceedings of the ACM International Conference on Multimedia.

[44] Altman NS (1992). An introduction to kernel and nearest-neighbor nonparametric regression. Am Stat.

[45] Rennie JD, Shih L, Teevan J, Karger DR. Tackling the poor assumptions of naive bayes text classifiers. International Conference on Machine Learning (ICML). 2003. p. 616–23.

[46] Cai Z, Xu L, Shi Y, Salavatipour MR, Goebel R, Lin G. Using gene clustering to identify discriminatory genes with higher classification accuracy. In: BioInformatics and BioEngineering, 2006. BIBE 2006. Sixth IEEE Symposium on; 2006. p. 235–42.

[47] Cho J-H, Lee D, Park JH, Lee I-B (2003). New gene selection method for classification of cancer subtypes considering within-class variation. FEBS Lett.

[48] Yuan Y, Shi Y, Su X, Zou X, Luo Q, Cai W, Han Z, Feng D (2017). Copy number aberration based cancer type prediction with convolutional neural networks. Proceedings of the 13th International Symposium on Bioinformatics Research and Applications (ISBRA 2017), Lecture Notes in Bioinformatics.

